# *Pseudomonas aeruginosa* isolates and their antimicrobial susceptibility pattern among catheterized patients at Jimma University Teaching Hospital, Jimma, Ethiopia

**DOI:** 10.1186/s13104-015-1497-x

**Published:** 2015-09-28

**Authors:** Temesgen Bekele, Amene Tesfaye, Tsegaye Sewunet, Habtewold Deti Waktola

**Affiliations:** Department of Pharmacy, College of Health Sciences, Jimma University, P. O. Box: 378, Jimma, Ethiopia; Department of Laboratory Sciences and Pathology, College of Health Sciences, Jimma University, Jimma, Ethiopia

**Keywords:** *Pseudomonas aeruginosa*, Urinary tract infection, Clinical isolates, Antimicrobial susceptibility

## Abstract

**Background:**

*Pseudomonas aeruginosa* is among the most common bacterial pathogens with wide spread distribution in health care settings. Despite advances in medical and surgical care and introduction of wide variety of antimicrobial agents, *Pseudomonas aeruginosa* continues to cause life threatening infection. Thus, this study aims to isolate and determine antimicrobial susceptibility patterns of *Pseudomonas aeruginosa* from catheterized patients with urinary tract infection.

**Result:**

A cross-sectional study was conducted from January to May, 2013. Urine specimens of 73 catheterized patients who developed urinary tract infection after catheterization were collected from sampling port of the catheter. The urine samples were inoculated on MaConckey and blood agar plates, and incubated at 37 °C for 24 h. The isolates were identified by conventional microbiological tests. Antimicrobial susceptibility pattern was determined by modified Kirby-Bauer disk diffusion method. From a total of 73 urine samples collected *P. aeruginosa* was isolated from 36 (49.32 %) catheterized patients; 17 (23.29 %) males and 19 (26.03 %) females. While all *P. aeruginosa* isolates were found to be susceptible to Norfloxacin and Ciprofloxacin most isolates were also susceptible to Gentamicin (86.12 %).

**Conclusion:**

The result shows higher prevalence of *P. aeruginosa* isolates among catheterized patients and the isolates were susceptible to the antimicrobials studied. All *P. aeruginosa* isolates were susceptible to Ciprofloxacin and Norfloxacin with some of the isolates shown resistance to Gentamicin. While the susceptibility of the isolates to the two fluoroquinolones is a good news for the prescribers their future rational prescription and use should be the main focus.

## Background

*Pseudomonas aeruginosa* is the third leading cause of hospital-acquired urinary tract infections (UTIs), accounting for about 12 % of all hospital acquired infections. It can invade the blood stream from the urinary tract, and this has been shown to be the source of nearly 40 % of *Pseudomonas* bacteremia. Urinary tract infection caused by *P. aeruginosa* are usually hospital-acquired and related to urinary tract catheterization instrumentation or surgery [1].

Infections caused by *Pseudomonas aeruginosa* are increasing both in hospital and in general community and it has been reported as one of the principal cause of nosocomial pathogen, particularly among immune compromised patient [1]. Concurrently, the extensive use of antimicrobial agents and the evolutionary antimicrobial resistance strategies of bacteria have resulted in the emergence of drug resistant bacteria. The efficiency of many antibiotics for treatment of infections has become quite limited due to the development of resistance and threat from antimicrobial resistant organisms is accumulating and accelerating [2].

*P.aeruginosa* was the second most common cause of pneumonia, the fourth most cause of urinary tract infection, and the sixth most common blood stream isolate in intensive care units (ICUs). Many potential reservoirs of infection have been identified in hospital environment, including respiratory equipment, cleaning solutions, disinfectants, sinks, vegetables, flowers, endoscopes, and physiotherapy pools [3].

The increasing prevalence of health-care associated infections (HAIs) produced by multidrug-resistant (MDR) *P. aeruginosa* strains severely compromises the selection of appropriate treatments and is therefore associated with significant morbidity and mortality [4]. Infections caused by *P. aeruginosa* are difficult to cure and often require combination therapy. For *P. aeruginosa*, antibiotic resistance is an increasing problem [5]. A varying degree of resistance to all known antipseudomonal antibiotics have been reported in different areas of the world by different authors [6–8]. Moreover, high rates of resistance to antibiotics are associated with nosocomial *P. aeruginosa* strains. It has been associated with sporadic or clustered cases of infection generally confined to single hospitalization units [8]. *P. aeruginosa* has been identified as the fourth most common causing catheter-associated urinary tract infections. The current increase in incidence of MDR isolates of *P. aeruginosa* raises serious concerns. Multidrug-resistance in *P. aeruginosa* is defined as the resistance to ≥3 of the following classes of antibiotics: penicillin’s/cephalosporin’s/monobactams, carbapenems, amino glycosides, and fluoroquinolones [9].

An alarming increase in the prevalence of MDR *P. aeruginosa* from 36 to 52 % were reported in an Egyptian study [3, 10]. In studies done in Pakistan and Iran, 29 and 30 % MDR phenotype was reported respectively [11].

## Methods

A cross-sectional study was conducted at Jimma University Teaching Hospital from Jan to May 2013. All catheterized patients found during the study period were conveniently enrolled in the study. The laboratory tests were performed at teaching laboratory of medical microbiology at Department of Laboratory Sciences and Pathology.

### Specimen collection, isolation and identification

Three to five milliliters of urine samples were collected through a sampling port (created just above where the storage bag tube fitted) from the catheter into clean and dry containers using 24 guage needle and syringe after cleaning the sampling port area with 70 % alcohol [12]. The specimens were transported immediately to the lab and inoculated on MaConckey and blood agar, and incubated aerobically at 37 °C for 24 h. Non-lactose fermenting pale colonies from MaConckey and large flat dark greenish colonies from blood agar (after sub-culturing on nutrient agar) were tested for conventional biochemical tests: oxidase test, catalase, citrate utilization, and oxidative fermentation. The isolates were further sub-cultured on to two nutrient agar plates and incubated separately (at 37 °C for pigment production and growth at 42 °C). *Pseudomonas aeruginosa* ATCC 27853 was used as a quality control strain.

Based on colony morphology, gram negative rod, oxidase positive, urease positive, Simon-citrates positive, and growth at 42 °C was confirmed as *P. aeruginosa*.

### Susceptibility tests

Antimicrobial susceptibility tests were done by the Kirby-Bauer disk diffusion method as per the recommendations of [14] against a panel of antipseudomonal antimicrobials of standard strengths. From each isolated pure colony inoculums were prepared in physiological saline by adjusting the turbidity of bacterial suspension to 0.5 McFarland’s standard; which is visually comparable to a microbial suspension of approximately 1.5 × 10^8^cfu/ml. Optimally, within 15 min after adjusting the turbidity of the inoculums suspension, a sterile cotton swab was dipped into the adjusted suspension. A dried surfaces of a Mueller–Hinton (Oxoid) agar plate were inoculated by spreading the swab over the entire sterile agar surface. This procedure is repeated by spreading two more times, rotating the plate approximately 60° each time to ensure an even distribution of inoculums [13]. Then the rim of the agar is swabbed. The following antibiotic discs were tested: Ciprofloxacin (5 µg), Gentamicin (10 µg), and Norfloxacin (10 µg) (Oxoid, Ltd., Basing stoke, Hampshire, England) standard antibiotic discs were placed on Muller–Hinton agar (Oxoid), which were previously inoculated with test strains and incubated at 37 °C for 16–18 h. After incubation, inhibition zones were recorded as the diameter of the clear zones around the disc and interpreted according to performance standard for antimicrobial disk susceptibility test (CLSI 2012) [14].

## Results

### Patients and specimens data

Of the total 73 catheterized patients recruited in this study 51 (69.68 %) were male and 22 (30.42 %) were females. Urine specimen of these patients were examined using standard laboratory procedure and a total of 36 (49.32 %) cultures were found positive for *P. aeruginosa*. Of these positive cultures for *P. aeruginosa,* 19 (26.03 %) and 17 (23.29 %) were identified from urine specimens of females and males respectively (Table 1). On the other hand; the proportion of positive cultures is very high among females (86.36) as compared to males (33.3). Most of the strains were isolated from older patients (≥45 years) (58.33 %) followed by 36–45 years old patients (22.32 %) (Table 2).

**Table 1:** Distribution of *Pseudomonas aeruginosa* isolates among males and females

Sex	No. tested (%)	No. positive (%)	No. negative (%)
Male	51 (69.86)	17 (33.33)	34 (66.67)
Female	22 (30.14)	19 (86.36)	3 (13.64)
Total	73 (100)	36 (49.32)	37 (50.68)

**Table 2:** Distribution of *Pseudomonas aeruginosa* among different age groups

Age group (years)	Number of isolates	Percentage (%)
14–25	4	11.12
26–35	3	8.33
36–45	8	22.32
>45	21	58.33
Total	36	100

### Antimicrobial susceptibility patterns

Antimicrobial susceptibility patterns of *P. aeruginosa* to the three drugs commonly used in our setup was examined. While all *P. aeruginosa* strains were susceptible to Norfloxacin and Ciprofloxacin most strains were also susceptible for Gentamicin (86.12 %) (Table 3).

**Table 3:** Antimicrobial susceptibility patterns of *Pseudomonas aeruginosa* isolates

Antibiotics	AST profile		
	Resistant (%)	Intermediate (%)	Susceptible (%)
Ciprofloxacin	–	–	36 (100)
Gentamicin	–	5 (13.88)	31 (86.12)
Norfloxacin	–	–	36 (100)

## Discussion

In spite of advances in medical care and infection prevention policies and practices; Catheter associated urinary tract infections (CAUTIs) remains a major problem. The increasing number of CAUTIs bears on the fact that urinary catheters became second most often used foreign body inserted into human body [15]. Several other studies [16, 17] have shown *P. aeruginosa* is the second most common gram negative microorganism isolated from catheterized patient with UTI. The prevalence of *P. aeruginosa* in the current study was found to be 49. 3 %. Compared to other similar studies (17 %) [16] the finding of this study is higher. It could be as a result of lack of consistent prescription policy and lack of reportable standard of care in our setup. In addition, duration of hospital stay and catheterization is also a major contributing factor [18, 19].

In this study higher proportion of females (86.36 %) were detected positive for *P. aeruginosa* than their male (33.3 %) counterparts while other studies reported higher prevalence of *P. aeruginosa* in males than in females [20, 21]. Nevertheless, it is comparable with the study from North Nigeria, where females found to be more infected by this bacterium [22]. Even though we did not control the study and thus cannot generate strong evidence from our data set physiological and anatomical factors might have played a role. The drier environment in the urethra, antimicrobial activity of prostate secretions and longer distance between the anus and urethra of males might delay or prevents the optimal growth of bacteria in males [23].

Another important factor in this study is age. The isolation rate was higher among old age greater than 45 years (58.33 %), and nearly 80.5 % of the isolates were from age group greater than 35 years. In light of this study advancing age might be one of the predisposing factor. Furthermore there are studies from Nigeria that reported similar observation. Thus it is wise to give due attention to UTI when catheterizing older patients.

Increasing resistance to different antipseudomonal drugs particularly among hospital strains has been reported world-wide. This is a serious therapeutic problem in the management of disease due to these organisms. In our case we tested susceptibility profiles of *P. aeruginosa* to commonly used antimicrobial agents in the area. The isolate were susceptible to Ciprofloxacin and Norfloxacin. The Susceptibility of the isolates to Ciprofloxacin is very important for local consumption compared to other studies, whereby 40.5 % [24], 50 % [17] and 72.41 % [25] susceptibility were observed, Ciprofloxacin should be given due attention and should be used when necessary as an alternative therapeutic agent for resistant isolates.

Aminoglycosides, especially gentamicin is a known frontline antibiotic in the treatment of bacterial infection by gram negative bacteria. However, emerging reports showed increased prevalence of resistance to these drugs. In this study, 13.88 % isolates were observed resistant for this drug. It is lower than a study conducted in Nigeria [26], in which all isolates were resistant. The fact that we do share similar threat in our hospital impose another point of concern.

## Conclusion

The result shows higher prevalence of *P. aeruginosa* isolates among catheterized patients where the proportion of female patients detected positive for *P. aeruginosa* was higher than their male counterparts. All *P. aeruginosa* isolates were susceptible to Ciprofloxacin and Norfloxacin with some of the isolates shown resistance to Gentamicin. While the susceptibility of the isolates to the two fluoroquinolones is a good news for the prescribers their future rational prescription and use should be the main focus.

## Abbreviations

AmpC: ampicillinase (β-lactamase enzyme)
AST: antimicrobial susceptibility testing
CAUTI: catheter associated urinary tract infections
CLSI: clinical laboratory standard institute
MDR: multidrug-resistant
UTI: urinary tract infections
